# Accelerated pentose utilization by *Corynebacterium glutamicum* for accelerated production of lysine, glutamate, ornithine and putrescine

**DOI:** 10.1111/1751-7915.12001

**Published:** 2012-11-20

**Authors:** Tobias M Meiswinkel, Vipin Gopinath, Steffen N Lindner, K Madhavan Nampoothiri, Volker F Wendisch

**Affiliations:** 1 Faculty of Biology & CeBiTec, Bielefeld UniversityD-33615, Bielefeld, Germany; 2 Biotechnology Division, National Institute for Interdisciplinary Science and Technology (NIIST)CSIR, Trivandrum, 695 019, Kerala, India

## Abstract

Because of their abundance in hemicellulosic wastes arabinose and xylose are an interesting source of carbon for biotechnological production processes. Previous studies have engineered several *Corynebacterium glutamicum* strains for the utilization of arabinose and xylose, however, with inefficient xylose utilization capabilities. To improve xylose utilization, different xylose isomerase genes were tested in *C. glutamicum*. The gene originating from *Xanthomonas campestris* was shown to have the highest effect, resulting in growth rates of 0.14 h^−1^, followed by genes from *Bacillus subtilis*, *Mycobacterium smegmatis* and *Escherichia coli*. To further increase xylose utilization different xylulokinase genes were expressed combined with *X. campestris* xylose isomerase gene. All combinations further increased growth rates of the recombinant strains up to 0.20 h^−1^ and moreover increased biomass yields. The gene combination of *X. campestris* xylose isomerase and *C. glutamicum* xylulokinase was the fastest growing on xylose and compared with the previously described strain solely expressing *E. coli* xylose isomerase gene delivered a doubled growth rate. Productivity of the amino acids glutamate, lysine and ornithine, as well as the diamine putrescine was increased as well as final titres except for lysine where titres remained unchanged. Also productivity in medium containing rice straw hydrolysate as carbon source was increased.

**Funding Information** No funding information provided.

## Introduction

Lignocellulosic hydrolysates contain glucose as well as significant amounts of the pentoses xylose (5–20%) and arabinose (1–5%) (Aristidou and Penttila, 2000). Lignocellulosic hydrolysates may be obtained from agricultural wastes such as rice straw and are therefore cheap carbon sources. However, lignocellulosic hydrolysates are not fully capitalized on since several industrially relevant microorganisms are not able to utilize pentose sugars as substrates (Jeffries and Jin, 2000). Metabolic engineering of pentose utilization has been successful in some cases, e.g. of *Saccharomyces cerevisiae*, while in other cases absent or inefficient pentose utilization is still a major bottleneck to be overcome for industrial processes based on lignocellulosic biomass (Aristidou and Penttila, 2000; Becker and Boles, 2003; Karhumaa *et al*., 2006; Hahn-Hagerdal *et al*., 2007a,2007b).

*Corynebacterium glutamicum* as a workhorse of industrial microbiology is well known for fermentative production of amino acids and has been engineered for the production of diamines like 1,4-diaminobutane (Schneider and Wendisch, 2010) and 1,5-diaminopentane (Mimitsuka *et al*., 2007; Kind *et al*., 2010; 2011), of ketoacids such as pyruvate (Wieschalka *et al*., 2012) and 2-ketoisovalerate (Krause *et al*., 2010), diacids such as succinate (Okino *et al*., 2008; Litsanov *et al*., 2012a,2012b,2012c) and the alcohols ethanol (Inui *et al*., 2004) and isobutanol (Blombach *et al*., 2011). Traditionally, technical substrates like starch hydrolysates and molasses are used in industrial processes. The respective sugars glucose (starch hydrolysate), fructose and sucrose (molasses) are taken up and are phosphorylated by the phosphoenolpyruvate-dependent carbohydrate phosphotransferase (PTS) system or, in the case of glucose, alternatively also by *myo*-inositol permeases with subsequent phosphorylation by ATP-and/or polyphosphate-dependent glucokinases (Lindner *et al*., 2010; 2011). The natural substrate spectrum of *C. glutamicum* further includes sugars like ribose or maltose, alcohols like ethanol or *myo*-inositol and organic acids like acetate, citrate, lactate, propionate and pyruvate and amino acids like l-glutamate (Kramer *et al*., 1990; Dominguez *et al*., 1998; Kiefer *et al*., 2002; Gerstmeir *et al*., 2003; Eikmanns, 2005; Moon *et al*., 2005; Polen *et al*., 2005; Stansen *et al*., 2005; Krings *et al*., 2006; Frunzke *et al*., 2008; Kato *et al*., 2010; Neuner and Heinzle, 2011). Within the flexible feedstock concept, the substrate spectrum of *C. glutamicum* has been extended by metabolic engineering to allow access to starch, cellobiose, lactose, galactose and glycerol as well as succinate, fumarate and malate as carbon sources (Brabetz *et al*., 1991; Cadenas *et al*., 1992; Kotrba *et al*., 2003; Barrett *et al*., 2004; Seibold *et al*., 2006; Tateno *et al*., 2007; Rittmann *et al*., 2008; Youn *et al*., 2008; 2009).

Similarly, *C. glutamicum* has been engineered for growth with the pentoses arabinose and xylose and for the production of ethanol, organic acids, amino acids and diamines from arabinose and/or xylose (Kawaguchi *et al*., 2006; 2008; Sasaki *et al*., 2009, 2010; Gopinath *et al*., 2011; Kind and Wittmann, 2011; Schneider *et al*., 2011). Metabolic engineering relied on bacterial pathway genes. In *Escherichia coli* and other bacteria able to utilize arabinose and/or xylose, arabinose is catabolized via arabinose isomerase (encoded by *araA*), ribulokinase (*araB*) and ribulose-5-phosphate-4-epimerase (*araD*) while xylose catabolism requires xylose isomerase (*xylA*) and xylulokinase (*xylB*) (Lin, 1996; Hahn-Hagerdal *et al*., 2007a,2007b). Heterologous expression of *araA, araB* and *araD* from *E. coli* resulted in *C. glutamicum* recombinants able to grow with arabinose as sole source of carbon (Kawaguchi *et al*., 2008). When the arabinose importer gene *araE* from *C. glutamicum* ATCC31831 was expressed in addition, faster growth with arabinose entailed (Sasaki *et al*., 2009). In the case of xylose, heterologous expression of a single *E. coli* gene, *xylA*, was sufficient to allow growth with xylose as sole carbon source (Kawaguchi *et al*., 2006) since the *C. glutamicum* genome encodes xylulokinase (Kalinowski *et al*., 2003). *Corynebacterium glutamicum* has proven a good choice for utilizing complex mixtures of carbon sources such as hemicellulosic hydrolysates because, unlike *E. coli* and *S. cerevisiae, C. glutamicum* efficiently co-utilizes different carbon sources when present in blends (Wendisch, 2006; Arndt and Eikmanns, 2008; Blombach and Seibold, 2010; Gopinath *et al*., 2011). Consequently, besides proof-of-concept using pure chemicals, growth and production with hemicellulosic hydrolysates obtained, e.g. from rice straw could be achieved (Gopinath *et al*., 2011). In the present study, we address xylose catabolism as a possible rate-limiting step of xylose-based production by *C. glutamicum*.

## Results

### Comparative analysis of recombinant *C. glutamicum* strains with different plasmid encoded xylose isomerases

*Corynebacterium glutamicum* possesses a xylulokinase and heterologous production of *E. coli* xylose isomerase allowed *C. glutamicum* to grow with xylose, however, the observed growth rates were low (0.09 h^−1^) as compared to growth rates, e.g. with glucose (0.32 h^−1^), ribose (0.23 h^−1^) or acetate (0.28 h^−1^) (Wendisch *et al*., 2000; Wendisch, 2003; Netzer *et al*., 2004). In order to test whether xylose isomerase activity is limiting growth with xylose of *C. glutamicum* recombinants expressing *xylA* from *E. coli*, several recombinants were constructed expressing xylose isomerase genes from different sources. The xylose isomerase genes of well-understood model organisms, plant pathogens and strains closely to *C. glutamicum* related *E. coli*, *Bacillus subtilis*, *Xanthomonas campestris* and *Mycobacterium smegmatis*, were cloned into the IPTG-inducible expression vector pEKEx3 and transformed into *C. glutamicum* WT (Table 1C). Xylose isomerase (XI) activity measured as described in *Experimental procedures* was not detectable in empty vector control strains (< 0.005 U mg^−1^) (Table 2). High and comparable XI activities were observed in crude extracts of WT(pEKEx3-*xylA_Ec_*) (0.095 ± 0.010 U mg^−1^) and WT(pEKEx3-*xylA_Xc_*) (0.090 ± 0.008 U mg^−1^), while about three times less activity was found for WT(pEKEx3-*xylA_Bs_*) (0.023 ± 0.003 U mg^−1^) and WT(pEKEx3-*xylA_Ms_*) (0.033 ± 0.007 U mg^−1^). While XI activity increase due to overexpression of *xylA* could be detected in each case, the values are difficult to compare as a single enzyme assay was used without optimization for the enzymes of various origin.

**Table 1:** List of sequences of oligonucleotide primers, plasmids and strains used

Name	Sequence (5′–3′) or function	Relevant characteristics or reference
**A. Oligonucleotides**		
xylB_fw_Bsu	GA**GAAAGGAGG**CCCTTCAG*ATG*AAGTATGTCATTGGAATAGATCTTGG	HE of Bsu *xylB*; **RBS**; *start*
xylB_rv_Bsu	GATCTAGA*TTA*GTTTTTTCGAAAGCTCTTCAAAGC	HE of Bsu *xylB*; XbaI; *stop*
xylB_fw_Cgl	GA**GAAAGGAGG**CCCTTCAG*ATG*GCTTTGGTTCTTGGAATCG	OE of Cgl *xylB*; **RBS**; *start*
xylB_rv_Cgl	GATCTAGA*CTA*GTACCAACCCTGCGTTG	OE of Cgl *xylB*; XbaI; *stop*
xylB_fw_Eco	GA**GAAAGGAGG**CCCTTCAG*ATG*TATATCGGGATAGATCTTGGCAC	HE of Eco *xylB*; **RBS**; *start*
xylB_rv_Eco	GATCTAGA*TTA*CGCCATTAATGGCAGAAGTTG	HE of Eco *xylB*; XbaI; *stop*
xylA_fw_Bsu	GATCTAGA**GAAAGGAGG**CCCTTCAG*ATG*GCTCAATCTCATTCCAGTTCA	HE of Bsu *xylA*; XbaI; **RBS**; *start*
xylA_rv_Bsu	GAGAGCTC*TTA*TACTTCTAAAATGTATTGGTTCAATATCGCT	HE of Bsu *xylA*; Ecl136II; *stop*
xylA_fw_Eco	GATCTAGA**GAAAGGAGG**CCCTTCAG*ATG*CAAGCCTATTTTGACCAGC	HE of Eco *xylA*; XbaI; **RBS**; *start*
xylA_rv_Eco	GAGAGCTC*TTA*TTTGTCGAACAGATAATGGTTTACCAG	HE of Eco *xylA*; Ecl136II; *stop*
xylA_fw_Msm	GATCTAGA**GAAAGGAGG**CCCTTCAG*ATG*ACCGTGTTGGAGTCGAA	HE of Msm *xylA*; XbaI; **RBS**; *start*
xylA_rv_Msm	GAGAGCTC*TCA*TCGCGCGCCCATCAG	HE of Msm *xylA*; Ecl136II; *stop*
xylA_fw_Xcc	GATCTAGA**GAAAGGAGG**CCCTTCAG*ATG*AGCAACACCGTTTTCATCG	HE of Xcc *xylA*; XbaI; **RBS**; *start*
xylA_rv_Xcc	GAGAGCTC*TCA*ACGCGTCAGGTACTGATT	HE of Xcc *xylA*; Ecl136II; *stop*
**B. Plasmids**		
pEKEx3	Spec^R^; *C. glutamicum*/*E. coli* shuttle vector (*P_tac_*, *lacI*^q^; pBL1, *OriV_Cg_*, *OriV_Ec_*)	Stansen *et al*. (2005)
pEKEx3-*xylA_Ec_*	Derived from pEKEx3, for regulated expression of *xylA_Ec_* (b3565) of *E. coli*	Gopinath *et al*. (2011)
pEKEx3-*xylA_Bs_*	Derived from pEKEx3, for regulated expression of *xylA_Bs_* (BSU17600) of *B. subtilis*	This work
pEKEx3-*xylA_Ms_*	Derived from pEKEx3, for regulated expression of *xylA_Ms_* (MSMEG_6021) of *M. smegmatis*	This work
pEKEx3-*xylA_Xc_*	Derived from pEKEx3, for regulated expression of *xylA_Xc_* (XCC1758) of *X. campestris*	This work
pEKEx3-*xylA_Xc_*-*xylB_Ec_*	Derived from pEKEx3, for regulated expression of *xylA_Xc_* (XCC1758) of *X. campestris* and *xylB_Ec_* (b3580) of *E. coli*	This work
pEKEx3-*xylA_Xc_*-*xylB_Bs_*	Derived from pEKEx3, for regulated expression of *xylA_Xc_* (XCC1758) of *X. campestris* and *xylB*_Bs_ (BSU17610) of *B. subtilis*	This work
pEKEx3-*xylA_Xc_*-*xylB_Cg_*	Derived from pEKEx3, for regulated expression of *xylA_Xc_* (XCC1758) of *X. campestris* and *xylB_Cg_* (cg0147) of *C. glutamicum*	This work
pVWEx1	Kan^R^; *C. glutamicum*/*E. coli* shuttle vector (*P_tac_*, *lacI*^q^; pHM1519, *OriV_Cg_*, *OriV_Ec_*)	Peters-Wendisch *et al*. (1998)
pVWEx1-*araBAD*	Derived from pVWEx1, for regulated expression of *araB* (b0063) and *araA* (b0062) and *araD* (b0061) of *E. coli*	Schneider *et al*. (2011)
**C. Strains**		
*E. coli*		
DH5α	F*thi-*1 *endA*1 *hsdr*17(r−, m−) *supE*44 _*lacU*169 (ф80*lac*Z_M15) *recA*1 *gyrA*96 *relA*1	Hanahan (1983)
*C. glutamicum*		
ATCC13032	Wild type (WT)	Kinoshita *et al*. (1957)
DM1729	*lysC* ^P458S^*, hom*^V59A^*, pyc* ^T311I^	Georgi *et al*. (2005)
ORN1	l-ornithine overproducing strain derived from ATCC13032, auxotrophic for l-arginine due to *argF* deletion	Schneider *et al*. (2011)
PUT21	ORN1 carrying pVWEx1-*speC*-5′_21_-*argF*	Schneider *et al*. (2011)

Restriction sites are underlined, ribosomal binding sites are shown in bold, stop and start codons are in italics.

OE, overexpression; HE, heterologous expression; RBS, ribosomal binding site; Cgl, *C. glutamicum*; Eco, *E. coli*; Bsu, *B. subtilis*; Msm, *M. smegmatis*; Xcc*, X. campestris*.

**Table 2:** Specific activities of different xylose isomerase and xylulokinase

	Specific activity (U mg^−1^ total protein)							
	WT(pEKEx3-x)							
	–	*xylA_Ec_*	*xylA*_Bs_	*xylA*_Ms_	*xylA*_Xc_	*xylA*_Xc_-*xylB_Ec_*	*xylA*_Xc_-*xylB*_Bs_	*xylA*_Xc_-*xylB_Cg_*
Xylose isomerase	< 0.005	0.095 ± 0.010	0.023 ± 0.003	0.033 ± 0.007	0.090 ± 0.008	0.062 ± 0.004	0.026 ± 0.003	0.077 ± 0.010
Xylulokinase	0.013 ± 0.005	0.020 ± 0.000	0.024 ± 0.000	0.019 ± 0.000	0.021 ± 0.000	0.541 ± 0.063	0.020 ± 0.004	0.468 ± 0.018

All tests were carried out with crude extracts at 30°C.

**Figure 1:**
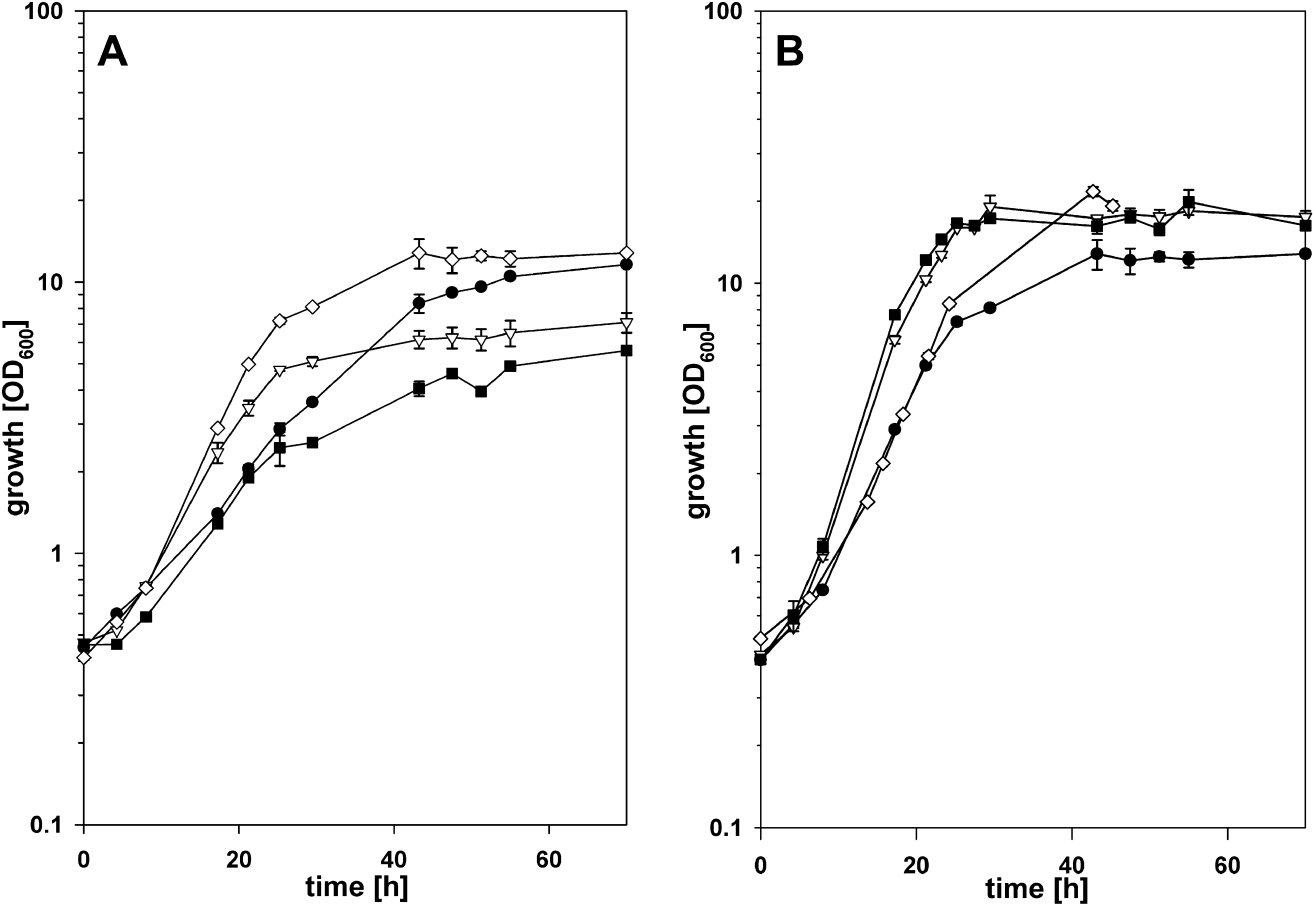
Growth of *C. glutamicum* strains in CgXII medium containing 100 mM xylose.A. *Corynebacterium glutamicum* strains WT(pEKEx3-*xylA_Xc_*) (open diamonds), WT(pEKEx3-*xylA_Bs_*) (open triangles), WT(pEKEx3-*xylA_Ec_*) (closed circles) and WT(pEKEx3-*xylA_Ms_*) (closed squares) were analysed.B. *Corynebacterium glutamicum* strains WT(pEKEx3-*xylA_Xc_-xylB_Bs_*) (open diamonds), WT(pEKEx3-*xylA_Xc_-xylB_Ec_*) (open triangles), WT(pEKEx3-*xylA_Xc_*) (closed circles) and WT(pEKEx3-*xylA_Xc_-xylB_Cg_*) (closed squares) were analysed. Data represents mean values and standard deviations of three independent cultivations.

To check the performance of the *C. glutamicum* strains harbouring the different XI genes growth experiments in CgXII minimal medium with 100 mM xylose as sole carbon source were performed (Fig. 1A). All recombinant strains expressing xylose isomerase genes were able to grow with xylose as sole carbon source (Fig. 1A). *Corynebacterium glutamicum* WT(pEKEx3-*xylA_Xc_*) showed the fastest growth (0.144 ± 0.001 h^−1^) and reached the highest biomass concentration (3.37 ± 0.12 gCDW l^−1^), followed by WT(pEKEx3-*xylA_Bs_*) (0.118 ± 0.007 h^−1^; 1.66 ± 0.15 gCDW l^−1^), WT(pEKEx3-*xylA_Ms_*) (0.093 ± 0.003 h^−1^; 1.29 ± 0.05 gCDW l^−1^) and WT(pEKEx3-*xylA_Ec_*) (0.090 ± 0.005 h^−1^; 2.79 ± 0.05 gCDW l^−1^). Thus, heterologous expression of the xylose isomerase gene from *X. campestris* improved xylose-utilization by recombinant *C. glutamicum* significantly reducing generation times from 7.7 h to 4.8 h.

### Comparative analysis of recombinant *C. glutamicum* strains overexpressing endogenous or heterologous xylulokinase genes

*Corynebacterium glutamicum* WT contains xylulokinase, however, xylulokinase activities determined as described in *Experimental procedures* were low in crude extracts of *C. glutamicum* WT, the empty vector control strain and of all recombinants expressing only a heterologous xylose isomerase gene (between 0.013 and 0.024 U mg^−1^) (Table 2). Ectopic expression *xylA* from *X. campestris* was combined either with overexpression of endogenous *xylB* or with overexpression of xylulokinase genes from *E. coli* or *B. subtilis* (Table 1C). Xylulokinase (XK) activity was not increased significantly in strain WT(pEKEx3-*xylA_Xc_*-*xylB_Bs_*) (Table 2). In contrast, ectopic expression of *xylB* from *E. coli* and overexpression of endogenous *xylB* increased XK activity in crude extracts about 25-fold. To test the effect of *xylB* overexpression in addition to *xylA* overexpression, growth of *C. glutamicum* strains overproducing the different XK's along with XI from *X. campestris* in CgXII minimal medium with 100 mM xylose as sole carbon source was compared (Fig. 1B). The control strain WT(pEKEx3-*xylA_Xc_*) reached a lower biomass concentration (3.37 ± 0.12 gCDW l^−1^) and grew with a slower growth rate (0.144 ± 0.001 h^−1^) than the strains overproducing XK gene in addition. *Corynebacterium glutamicum* WT(pEKEx3-*xylA_Xc_-xylB_Cg_*) grew fastest (0.199 ± 0.009 h^−1^) and reached the highest biomass concentration (4.87 ± 0.53 gCDW l^−1^) followed by WT(pEKEx3-*xylA_Xc_-xylB_Ec_*) (0.189 ± 0.001 h^−1^; 4.82 ± 0.33 gCDW l^−1^) and WT(pEKEx3-*xylA_Xc_-xylB_Bs_*) (0.162 ± 0.001 h^−1^; 5.30 ± 0.22 gCDW l^−1^). Thus, heterologous expression of the endogenous xylulose kinase gene from *C. glutamicum* in addition to the xylose isomerase gene from *X. campestris* further improved xylose utilization significantly reducing generation times from 4.8 h to 3.5 h.

### Amino acid and diamine production from xylose by the improved strain

**Figure 2:**
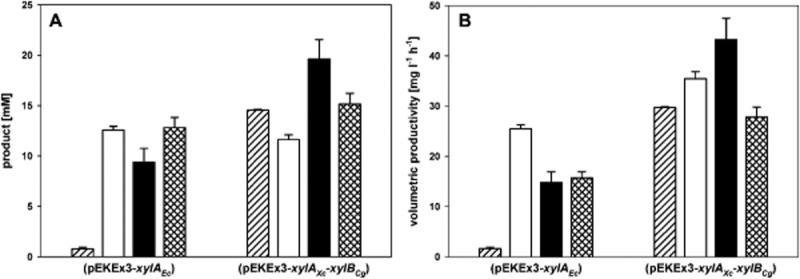
Product concentrations (A) and volumetric productivities (B) for l-glutamate, l-lysine, l-ornithine and putrescine production in CgXII medium containing 100 mM xylose. *Corynebacterium glutamicum* strains with pEKEx3-*xylA_Ec_* or pEKEx3-*xylA_Xc_-xylB_Cg_* were analysed. l-glutamate was produced with WT (hatched bars), l-lysine with DM1729 (open bars), l-ornithine with ORN1 (closed bars) and putrescine with PUT21 (checked bars). Data represent mean values and experimental imprecision of two independent cultivations.

Previously, we showed production of amino acids like l-glutamate and l-lysine as well as the diamine putrescine from xylose minimal medium by strains harbouring the basic xylose utilization plasmid pEKEx3-*xylA_Ec_* (Gopinath *et al*., 2011). The improved plasmid pEKEx3-*xylA_Xc_*-*xylB_Cg_* was transformed into the model lysine producer DM1729, the model ornithine producer ORN1 and the model 1,4-diaminobutane producer PUT21. l-glutamate production in CgXII minimal medium with 100 mM xylose as sole carbon source by *C. glutamicum* WT(pEKEx3-*xylA_Ec_*) and by WT(pEKEx3-*xylA_Xc_*-*xylB_Cg_*) was triggered by ethambutol addition and the improved strain reached higher titres (14.5 ± 0.1 mM as compared with 0.8 ± 0.1 mM) and exhibited an increased productivity (29.7 ± 0.2 as compared with 1.6 ± 0.3 mg l^−1^ h^−1^, Fig. 2). Lysine production by DM1729(pEKEx3-*xylA_Xc_*-*xylB_Cg_*) was characterized by a volumetric productivity improved from 25.5 ± 0.8 to 35.4 ± 1.4 mg l^−1^ h^−1^. The volumetric ornithine productivity by ORN1(pEKEx3-*xylA_Xc_*-*xylB_Cg_*) was higher than that of the control (43.2 ± 4.3 as compared with 14.8 ± 2.2 mg l^−1^ h^−1^) and higher ornithine concentrations were achieved (19.6 ± 1.9 mM as compared with 9.4 ± 1.4 mM). Also putrescine production was faster (27.8 ± 2.0 as compared with 15.7 ± 1.2 mg l^−1^ h^−1^) and titres rose from 12.9 ± 1.0 mM to 15.1 ± 1.1 mM. Taken together, all recombinants carrying the improved plasmid pEKEx3-*xylA_Xc_*-*xylB_Cg_* showed significantly increased volumetric productivities in medium with pure xylose.

### Amino acid production on rice straw hydrolysate

**Figure 3:**
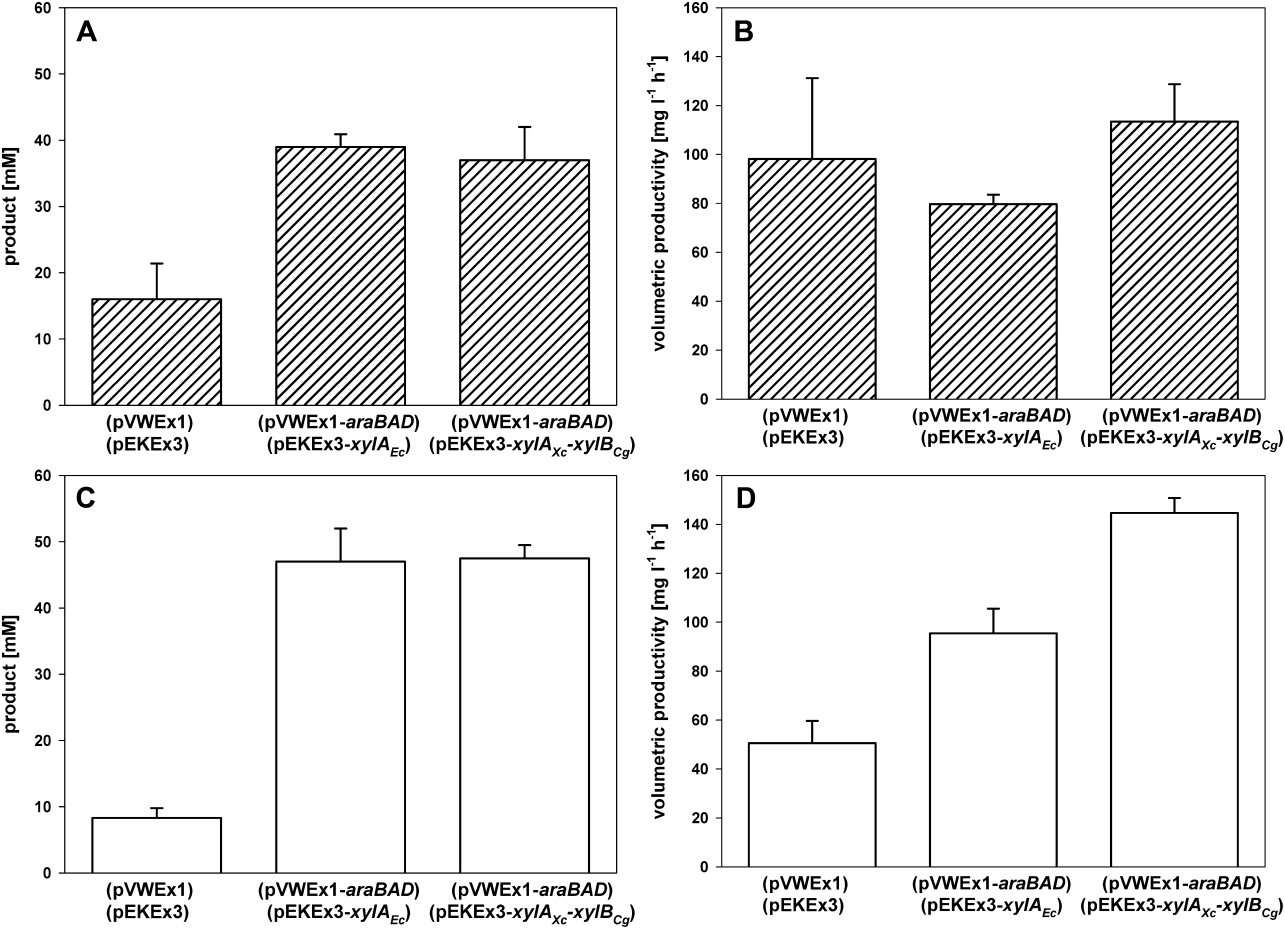
Product concentrations (A, C) and volumetric productivities (B, D) for l-glutamate (A, B) and l-lysine (C, D) production in CgXII medium containing rice straw hydrolysate. *Corynebacterium glutamicum* strains with empty vectors, pVWEx1-*araBAD* and pEKEx3-*xylA_Ec_* or pVWEx1-*araBAD* and pEKEx3-*xylA_Xc_-xylB_Cg_* were analysed. l-glutamate was produced with WT (hatched bars) and l-lysine with DM1729 (open bars). Data represent mean values and experimental imprecision of two independent cultivations.

To characterize l-glutamate and l-lysine production from hemicellulosic hydrolysates in particular rice straw hydrolysate (52 mM glucose, 203 mM xylose, 55 mM arabinose) derivatives of *C. glutamicum* WT or l-lysine model producer DM1729 harbouring either empty vectors, pVWEx1-*araBAD* and pEKEx3-*xylA_Ec_* or pVWEx1-*araBAD* and pEKEx3-*xylA_Xc_*-*xylB_Cg_* were used. The empty vector control utilized glucose for biomass formation and amino acid production, while the pentose-utilizing recombinants grew to higher biomass concentrations and produced more l-glutamate and l-lysine as they utilized arabinose and xylose in addition to glucose (Fig. 3). In case of l-glutamate the empty vector control reached 16 ± 5.4 mM and a volumetric productivity of 98.1 ± 33.1 mg l^−1^ h^−1^ in contrast to the pentose-utilizing strain WT(pVWEx1-*araBAD*)(pEKEx3-*xylA_Ec_*) with 39 ± 1.9 mM l-glutamate and a productivity of 79.7 ± 3.9 mg l^−1^ h^−1^. The strain improved for xylose utilization reached a comparable level of l-glutamate at 37 ± 5 mM and the highest productivity at 113.4 ± 15.3 mg l^−1^ h^−1^. As expected for growth-coupled l-glutamate production the specific productivities were similar [around 2.6, 3.0 and 3.1 mg gCDW^−1^ h^−1^ for the empty vector control, WT(pVWEx1-*araBAD*)(pEKEx3-*xylA_Ec_*) and WT(pVWEx1-*araBAD*)(pEKEx3-*xylA_Xc_*-*xylB_Cg_*) respectively].

As observed in the l-glutamate production experiment, product formation and productivity strongly depends on the ability to utilize the pentose fraction of the rice straw hydrolysate due to the expression of *araBAD* and *xylA* and/or *xylA* along with *xylB*. Therefore empty vector control reached 8.3 ± 1.5 mM l-lysine and a volumetric productivity of 50.6 ± 9.1 mg l^−1^ h^−1^ in contrast to the pentose-utilizing strain DM1729(pVWEx1-*araBAD*)(pEKEx3-*xylA_Ec_*) with clearly increased 47 ± 5 mM l-lysine and a productivity of 95.4 ± 10.2 mg l^−1^ h^−1^. The strain improved for xylose utilization reached a similar level of l-lysine at 47.5 ± 2 mM together with the highest productivity at 144.7 ± 6.1 mg l^−1^ h^−1^. As l-lysine production was growth-coupled the specific productivities were similar [around 2.6, 3.0 and 3.1 mg gCDW^−1^ h^−1^ for the empty vector control, DM1729 (pVWEx1-*araBAD*)(pEKEx3-*xylA_Ec_*) and DM1729 (pVWEx1-*araBAD*)(pEKEx3-*xylA_Xc_*-*xylB_Cg_*) respectively].

## Discussion

The newly engineered strain WT(pEKEx3-*xylA_Xc_*-*xylB_Cg_*) was shown to grow significantly faster (0.199 ± 0.009 h^−1^) on minimal medium containing xylose as sole carbon source compared with the previously described strain WT(pEKEx3-*xylA_Ec_*) (0.090 ± 0.005 h^−1^) (Gopinath *et al*., 2011) expressing *xylA* from *E. coli* only. A first improvement was already achieved by expressing different *xylA* genes, where *xylA* from *X. campestris* performed best (0.144 ± 0.001 h^−1^). By additional production of xylulokinase from different organisms further growth acceleration was observed with the fastest growing strain mentioned above. These findings let to the construction of production strains for lysine, glutamate, ornithine and putrescine for optimized utilization of xylose. The newly engineered xylose utilization strains showed a significantly higher volumetric productivity (l-glutamate: 29.7 ± 0.2 mg l^−1^ h^−1^; l-lysine: 35.4 ± 1.4 mg l^−1^ h^−1^; l-ornithine: 43.2 ± 4.3 mg l^−1^ h^−1^; putrescine: 27.8 ± 2.0 mg l^−1^ h^−1^) compared with production strains using the previously reported (Gopinath *et al*., 2011) xylose utilization plasmid (l-glutamate: 1.6 ± 0.3 mg l^−1^ h^−1^; l-lysine: 25.5 ± 0.8 mg l^−1^ h^−1^; l-ornithine: 14.8 ± 2.2 mg l^−1^ h^−1^; putrescine: 15.7 ± 1.2 mg l^−1^ h^−1^). Also during growth and production on rice straw hydrolysate a clear increase in volumetric productivity was observed for strains carrying pEKEx3-*xylA_Xc_*-*xylB_Cg_* (l-glutamate: 113.4 ± 15.3 mg l^−1^ h^−1^; l-lysine: 144.7 ± 6.1 mg l^−1^ h^−1^) compared with strains with pEKEx3-*xylA_Ec_* (l-glutamate: 79.7 ± 3.9 mg l^−1^ h^−1^; l-lysine: 95.4 ± 10.2 mg l^−1^ h^−1^) and in case of lysine production as well compared with the empty vector control strain, which is only capable of utilizing the glucose part of rice straw hydrolysate. As expected for growth-coupled amino acid production the specific productivities normalized to the biomass concentrations were similar.

Engineering for a better use of second-generation feedstock like rice straw hydrolysate, one possible bottleneck regarding catabolism of those carbon sources was successfully dealt with in this work. Further possible bottlenecks are transport of the carbon sources into the cell and the process of breaking the poly-and oligomeric sugars into their monomeric compounds to make them accessible for the producing microorganisms. Concerning the later point in this study a mild sulfuric acid treatment was used to hydrolysate the rice straw (Gopinath *et al*., 2011). A potential formation of typical fermentation inhibitors, e.g. 5-HMF or weak acids, may result in slower growth and lower production (Palmqvist *et al*., 1999; Zaldivar and Ingram, 1999; Zaldivar *et al*., 1999; 2000; Klinke *et al*., 2004; Heer and Sauer, 2008; Gopinath *et al*., 2011) and could be an aim to analyse in more detail in future studies for further optimization. However it was already described that in case of ethanol production by growth-arrested cells, typical inhibitors like organic acids, phenolic inhibitors or furans did not substantially disturb *C. glutamicum* (Sakai *et al*., 2007). In principal overcoming inhibition can be achieved by different ways, e.g. by simple resistance to the inhibitory substances due to efflux pump or prevention of uptake, by degradation of the relevant substances (Koopman *et al*., 2010) or by simply not creating inhibitors during processing of the substrates.

Dealing with the potential bottleneck of transport, the xylose and/or arabinose transporting system in the used *C. glutamicum* wild-type strain ATCC13032 is still unknown and therefore the heterologous expression of xylose and/or arabinose transport systems, e.g. *araE* (Sasaki *et al*., 2009), might result in faster substrate uptake and also higher productivity. AraE from *C. glutamicum* ATCC31831 might be a promising target for transport optimization because this uptake system accepts both arabinose and xylose and allows growth on even very low arabinose or xylose concentrations (Sasaki *et al*., 2009) and the donor strain is closely related to the used *C. glutamicum* ATCC13032.

With the potential for further improvements this study has clearly shown the ability of the already industrially intensively used *C. glutamicum* (Eggeling and Bott, 2005; Wendisch, 2006) to play a key role in utilization of second-generation feedstocks with respect to a wide product spectra reaching from products like amino acids to products like fine chemicals as diamines, e.g. putrescine.

## Experimental procedures

### Microorganisms and cultivation conditions

*E. coli* strain DH5α (Hanahan, 1985) was used for cloning and was cultivated in lysogeny broth medium (LB) (Sambrook *et al*., 1989). *Corynebacterium glutamicum* strains used in this work are wild-type strain ATCC13032 (WT) (Abe *et al*., 1967), l-lysine producing model strain DM1729 (Georgi *et al*., 2005), l-ornithine producing strain ORN1 and putrescine producing strain PUT21 (Schneider *et al*., 2011). Pre-cultures of *C. glutamicum* strains were inoculated from brain heart infusion (BHI) plates into BHI medium. For growth experiments with *C. glutamicum* 50 ml of BHI overnight cultures were harvested by centrifugation (10 min; 3220 *g*), washed in CgXII (Eggeling and Bott, 2005), centrifuged again and inoculated in CgXII medium to a final optical density (λ = 600 nm) (OD_600_) of 0.5. All growth and production experiments were carried out with CgXII medium in baffled shake flasks at 30°C and 120 r.p.m. When appropriate 100 μg ml^−1^ spectinomycin, 25 μg ml^−1^ kanamycin and 1 mM isopropyl-β-d-thiogalactopyranosid (IPTG) were added to the medium. l-glutamate excretion was triggered by addition of 500 μg ml^−1^ ethambutol (Radmacher *et al*., 2005). Ethambutol triggering of glutamate production was preferred over triggering by biotin limitation to avoid effects of residual biotin in hydrolysates. ORN1 was supplemented by addition of 500 μM l-arginine to minimal medium (Schneider *et al*., 2011). Growth was followed by OD_600_ determination until the stationary phase. OD_600_ was measured using a UV-1650 PC photometer (Shimadzu, Duisburg, Germany) in dilutions resulting in an OD_600_ between 0.05 and 0.25. The plasmids used in this study are listed in Table 1B.

### Heterologous expression of *xylA* and *xylB* genes from *B. subtilis*, *E. coli*, *M. smegmatis* and *X. campestris* and overexpression of *xylB* from *C. glutamicum*

For heterologous expression of genes encoding xylose isomerase (*xylA*) and xylulokinase (*xylB*) from *B. subtilis, E. coli, M. smegmatis* and *X. campestris* the genes were amplified via PCR from genomic DNA of *E. coli* MG1655, *B. subtilis* strain 168, *M. smegmatis* MC2 155 and *X. campestris* pv. campestris ATCC33913.

DNA from of *E. coli*, *B. subtilis, M. smegmatis* and *X. campestris* was prepared by using DNA isolation Kit (Roche, Mannheim, Germany). For overexpression of *xylB*, the gene was amplified via PCR from genomic DNA of *C. glutamicum* WT, which was prepared as described previously (Eikmanns *et al*., 1995).

Genes were amplified by PCR using the oligonucleotide primer pairs xylA_fw_Eco and xylA_rv_Eco, xylA_fw_Bsu and xylA_rv_Bsu, xylA_fw_Msm and xylA_rv_Msm, xylA_fw_Xcc and xylA_rv_Xcc, xylB_fw_Eco and xylB_rv_Eco, xylB_fw_Bsu and xylB_rv_Bsu and xylB_fw_Cgl and xylB_rv_Cgl (oligonucleotide sequences are listed in Table 1A). PCR products encoding XylA were cloned blunt into *SmaI*-restricted vector pEKEx3 (Stansen *et al*., 2005) and *xylB* genes were cloned into *Ecl136*II-restricted vector pEKEx3-*xylA_Xc_* resulting in the pEKEx3 derivatives listed in Table 1B, pEKEx3 allows IPTG-inducible gene expression. All resulting vectors were sequenced to confirm their sequence integrity.

### Enzyme activity measurements

Enzyme activity measurements were analysed in crude extracts of *C. glutamicum* (Guyer *et al*., 1981). Cells were inoculated from LB overnight cultures to an OD_600_ of 0.5 in 50 ml of LB medium containing 1 mM IPTG. Cells were harvested by centrifugation at a final OD_600_ of 4 and stored at −20°C until use.

Xylose isomerase and xylulokinase activity was measured by the determination of NADH using sorbitol dehydrogenase in the case of xylose isomerase and pyruvate kinase as well as lactate dehydrogenase in case of xylulokinase. Xylose isomerase assays were carried out at 30°C in a total volume of 1 ml containing 100 mM TRIS/HCl, pH 7.5, 10 mM MgCl_2_, 0.23 mM NADH and sorbitol dehydrogenase (1 U) (Brat *et al*., 2009), in the case of xylulokinase the assay contained pyruvate kinase (6.8 U), lactate dehydrogenase (9.9 U), 2 mM phosphoenolpyruvate, 0.2 mM NADH, 1 mM ATP, 2 mM MgCl_2_ and 50 mM TRIS/HCl, pH 7.5 (Eliasson *et al*., 2000). Tests were started by addition of d-xylose (2 M) or d-xylulose (167 mM) respectively. Enzymatic activities are displayed in μmol min^−1^ mg^−1^, defined as one unit (U). Continuous measurements were carried out using a Shimadzu UV-1650 PC photometer (Shimadzu, Duisburg, Germany).

Protein concentrations in crude extracts were determined using Bradford reagents (Sigma, Taufkirchen, Germany) and concentrations were calculated against bovine serum albumin standards.

### Acid hydrolysis of agricultural residues

Hydrolysis of rice straw has been carried out as described before (Gopinath *et al*., 2011).

### Determination of amino acid and diamine concentrations

Amino acids l-lysine, l-glutamate and l-ornithine were quantified via HPLC as described previously (Georgi *et al*., 2005). Putrescine was quantified via HPLC as described before (Schneider *et al*., 2011).

## Conflict of interest

None declared.
